# Book Reviews

**Published:** 1872-09-01

**Authors:** 


### Book Reviews.

A Manual of Qualitative Analysis. By Robert Galloway, F.R.C.S. From the fifth re-written and enlarged London edition. H. C. Lea, Philadelphia. Chicago: W. B. Keen & Cooke.

This is a small volume of 390 pages, and contains numerous illustrations. It is a work already extensively and favorably known through its former editions.

Hysterology. A Treatise, Descriptive and Clinical, on the Diseases and Displacements of the Uterus. By Edwin Nesbit Chapman, M.A., M.D. New York: Wm. Wood & Co., Publishers. Chicago, for sale by Jansen, McClurg & Co.

A volume of 500 pages, published in good style and binding. The subject is treated under the following headings: "Symptoms and Examination"—"Anatomy and Physiology"—"Pathology and Œtiology"—"Nulliparæ and Multiparæ"—"Active Conjestion of Uterus"—"Passive Congestion of Uterus"—"Treatment."

A Manual of Chemical Physiology, including its points of contact with Pathology. By J. L. W. Thudicham, M.D. Published by Wm. Wood & Co., New York. For sale by Jansen, McClurg & Co., Chicago.

This little work forms a complete and concise epitome of the branch cf science commonly termed physiological or animal chemistry, including its latest acquisitions. As the author states in the preface, it offers to the student in chemistry, physiology or science, a ready help to the acquisition of elementary knowledge, upon the basis of which he can afterwards place the superstructure of more extended and detailed studies. To the medical profession it will afford an easy bird's-eye view of the chemical features of the field of their thoughts and actions. The second part of the work is an analytical guide for the use of those who desire to make themselves practically acquainted with the phenomena and constituents of animal bodies.

The treatise summarizes much of the method pursued, and many of the results arrived at by the author during many years of patient labor and inquiry.

